# The intention to use HIV-pre-exposure prophylaxis (PrEP) among men who have sex with men in Switzerland: testing an extended explanatory model drawing on the unified theory of acceptance and use of technology (UTAUT)

**DOI:** 10.1007/s10389-017-0869-1

**Published:** 2017-11-22

**Authors:** Sibylle Nideröst, Daniel Gredig, Benedikt Hassler, Franziska Uggowitzer, Patrick Weber

**Affiliations:** University of Applied Sciences and Arts Northwestern Switzerland, School of Social Work, Riggenbachstrasse 16, 4600 Olten, Switzerland

**Keywords:** HIV/AIDS, Pre-exposure prophylaxis (PrEP), Men having sex with men (MSM), Acceptability, Unified theory of acceptance and use of technology, Switzerland

## Abstract

**Aim:**

The aim of this study was to determine the intention to use pre-exposure prophylaxis (PrEP) when available and to identify predictors of the intention to use PrEP among men who have sex with men (MSM) living in Switzerland. The theoretical model drew on the Unified Theory of Acceptance and Use of Technology and considered additional variables related specifically to PrEP, HIV protection and the resources of MSM.

**Subject and methods:**

For data collection, we used an anonymous, standardized self-administered online questionnaire. In 2015, we gathered a convenience sample of 556 HIV-negative MSM living in Switzerland. We analyzed the data using descriptive and bivariate statistics and used structural equation modeling to test the hypothesized model.

**Results:**

Predictors of respondents’ moderate intention to use PrEP were performance expectancy, effort expectancy, perceived social influence, concerns about using PrEP, attitudes toward condom use, negative experiences of condom use and age. These variables were predicted by HIV protection-related aspects and resources.

**Conclusion:**

The findings provide insights into the complex dynamic underlying the intention to use PrEP.

## Introduction

As in other countries, and since the beginning of the HIV epidemic, in Switzerland men having sex with men (MSM) have been the group most affected by HIV infections. After a rebound from 2003 to 2008, the number of newly diagnosed HIV infections in MSM declined, plateauing at a level still higher than 2003 (Bundesamt für Gesundheit 2010). Also, in recent years, between 40% and 50% of new HIV diagnoses continued to be in MSM. This reflects the finding that globally MSM are disproportionally affected by HIV (Beyrer et al. 2012). In 2015, 65% of the newly detected infections among MSM were classified as recent (Bundesamt für Gesundheit 2016), which is interpreted as an expression of an ongoing epidemic in this population (Bundesamt für Gesundheit 2008).

For populations most at risk for HIV infection, such as MSM, pre-exposure prophylaxis (PrEP) is considered an additional new option in combination HIV prevention. A number of trials have shown the efficacy of daily oral PrEP with tenofovir/emtricitabine (Truvada®) in significantly reducing the HIV infection risk among those adhering to the prescription, both among MSM (Grant et al. 2010; Liu et al. 2016; Yang et al. 2013) and other groups (Baeten et al. 2012; Riddell and Cohn 2016; Thigpen et al. 2012). More recent trials showed that ‘on-demand’ or ‘event-based dosing’ (oral use of PrEP before and after sex) also yielded high levels of efficacy among MSM (McCormack et al. 2016; Molina et al. 2015). Findings from demonstration projects in the USA suggest that the effectiveness of PrEP in MSM can also be assumed when dispensed in the settings of STI clinics and community health services (Liu et al. 2016).

The demonstrated efficacy prompted the call to fully embrace PrEP and scale up the use of this prevention method (Beyrer et al. 2015). In 2012, the USA was the first country to approve PrEP and to issue guidelines for daily oral use of tenofovir/emtricitabine. The World Health Organization (WHO) included PrEP for MSM in the guidelines on HIV prevention in 2014 (World Health Organization 2014). Since then, a growing number of countries including France, Kenya, South Africa, Canada, Australia and, more recently, the European Union have approved the prescription of tenofovir/emtricitabine for PrEP while not necessarily integrating PrEP in their public insurance schemes or providing public cost cover.

However, the uptake of PrEP seems to be slow, also among MSM who are considered to benefit most from this new prevention option (Kirby and Thornber-Dunwell 2014). Data from the USA where PrEP was available first strengthen the assumption that HIV-negative MSM seem to be reluctant to adopt PrEP. In 2012, among a sample of black MSM living in Atlanta (USA), 7% reported currently taking PrEP (Eaton et al. 2014). The Annual American Men’s Internet Survey suggests that only 2.8% of eligible MSM reported ever having used PrEP (Grov et al. 2015). A more recent study showed that in late 2014 and early 2015 a proportion of 4.9% of the participating MSM largely living in major US cities had used PrEP in the previous 12 months (Delaney et al. 2016). Studies from other countries seem to point in the same direction (Hugo et al. 2016).

Apparently, there is a gap between the use and the reported willingness to use PrEP. The reported proportion of MSM willing to use PrEP is markedly higher. Although it varies considerably among studies, the reported willingness to use PrEP ranges from 19.1% (Ding et al. 2016) to 96.2% (Peinado et al. 2013). The heterogeneity of findings seems to be due to methods, region, population and recruitment venue. A recent comprehensive meta-analysis found that the global overall acceptability among MSM was 57.8%. This proportion was assessed as moderate (Peng et al. 2017). Studies on the motivation and willingness to use PrEP among MSM living in Australia that were carried out before PrEP was approved found that, in 2011, 28.2% of the respondents were willing to use PrEP (Holt et al. 2012) and, in late 2012, 26% of the participants were likely and 10.7% very likely to use PrEP as soon as it would become available in Australia (Prestage et al. 2014). Regarding Europe, there was scant knowledge about the acceptability of PrEP among MSM as evidences the overview by Young and McDaid (Young and McDaid 2014). However, recent studies on the intention to use daily oral PrEP among MSM revealed that 50.3% of a sample of MSM living in London (Aghaizu et al. 2013) and 47.8% of a sample of MSM living in Scotland reported that they would likely use PrEP if it were available (Frankis et al. 2016).

Nevertheless, these findings confirm certain reservations regarding PrEP. To fully integrate this new prevention option into the public health response to HIV and AIDS and to have it play the intended substantial role in future HIV prevention (Simpson and Gumel 2017), it will be crucial to gain knowledge not only about the awareness of PrEP (see, for example, Eaton et al. 2015), but also about the intention to use this new HIV prevention option in key populations; it will be important to understand the decision making of potential users and to elicit the dynamics influencing the intention to use PrEP (Auerbach and Hoppe 2015; UNAIDS 2014).

Since PrEP is globally discussed as an additional HIV prevention method, its acceptability among different target groups has been the subject of numerous studies (see, for example, Barash and Golden 2010; Krakower et al. 2012; Leonardi et al. 2011; Mimiaga et al. 2009). Some qualitative studies explored the reasoning regarding using PrEP of people supposed to take advantage of PrEP, such as MSM (Brooks et al. 2011; Pérez-Figueroa et al. 2015). To obtain an in-depth understanding of the dynamics underlying the decision-making of MSM in favor or against the use of PrEP, however, comprehensive explanatory models will be essential.

In Switzerland, where PrEP has not yet been approved and has only been used in an off-label trial with serodiscordant heterosexual couples (Vernazza et al. 2011), little is known about the intention to use PrEP in MSM. In 2015, a qualitative focus group study provided initial insights, showing that the acceptability of PrEP varied considerably among the participants (Gredig et al. 2016). However, the study design did not allow for conclusions on the prevalence of these conceptions of PrEP use or for identifying the contribution the identified factors might make to the explanation of the participants’ stated intention to use PrEP. In 2017, a brief survey of a sample of MSM with a profile on the online dating platform Grindr® established that 4.3% of the participants were using PrEP and 49.9% reported that they would consider using PrEP in the next 6 months (Hampel et al. 2017). However, this survey did not explore respondents’ reasons for using PrEP. Therefore, it did not add to the understanding of the dynamics underlying the intention to use this prevention option. Before implementing PrEP in Switzerland, knowledge about its acceptability and factors influencing the intention to use it are of interest from a public health and community perspective as well as from an economic and political point of view.

Against this background, the objectives of the study were (1) to determine the intention to use PrEP among HIV-negative MSM living in Switzerland, (2) to explain their intention to use PrEP, when available, and, therefore, (3) test an explanatory model of the intention to use PrEP.

### Modeling acceptance and intention to use PrEP: The theoretical framework

When we set out to model the intention to use PrEP among MSM, we chose to draw on specific models proven to be empirically effective in explaining the acceptance and use of other new technologies available. Thus, in defining the theoretical framework of this study, we built on the *Unified Theory of Acceptance and Use of Technology* (UTAUT) (Venkatesh et al. 2003). This model, developed by Venkatesh et al. (2003), combines elements of the theory of planned behavior (Ajzen 1991) and variables from previous technology acceptance models (Davis 1989). The UTAUT posits that the use of a specific technology is directly predicted by the intention to use this technology and facilitating conditions. The intention, in turn, is directly predicted by both performance expectancy and effort expectancy toward the technology in question and by social influence. Moreover, age, gender, experience and voluntariness of use act as moderators.

For this study, we specified and modified this model to fit the Swiss context: As PrEP is not available in Switzerland, we specified the model to explain the intention to use PrEP and excluded the variable ‘experiences’ in using PrEP. Given that the study focused exclusively on men, we dismissed the variable ‘gender.’ At the same time, we included variables that had proved to be predictors of PrEP use in previous studies and were corroborated by the findings of a qualitative focus group study regarding the intention to use PrEP in MSM living in Switzerland (Gredig et al. 2016). Thus, costs (Galea et al. 2011; Golub et al. 2013; Mimiaga et al. 2009; Smith et al. 2012) were integrated into effort expectancy, and the expected effectiveness of PrEP (Galea et al. 2011; Nodin et al. 2008; Smith et al. 2012) was considered in the performance expectancy. We added concerns, which included worries about possible negative side effects (Galea et al. 2011; Golub et al. 2013; Mimiaga et al. 2009; Saberi et al. 2012; Smith et al. 2012) and anticipated PrEP-related stigma (Ayala et al. 2013; Elst et al. 2013; Smith et al. 2012). Furthermore, we considered HIV-protection-related aspects as HIV risk behavior, negative experiences of condom use, attitudes toward condom use (Brooks et al. 2012; Eisingerich et al. 2012; Holt et al. 2012; Leonardi et al. 2011) and having been treated for a sexually transmitted infection (STI). We also kept age (Eisingerich et al. 2012; Holt et al. 2012; Krakower et al. 2012) and included further resources as income (Barash and Golden 2010; Eisingerich et al. 2012; Holt et al. 2012), education (Khawcharoenporn et al. 2012; Mimiaga et al. 2009; Zhang et al. 2013) and another aspect known to influence HIV-protective behavior, namely the type of somatic culture men have adopted (Gredig et al. 2007). Somatic culture refers to a system of deeply internalized rules and implicit schemata adopted in the socialization process that mold a person’s relationship, perception, thinking and acting regarding his or her own body (Boltanski 1976).

**Fig. 1:**
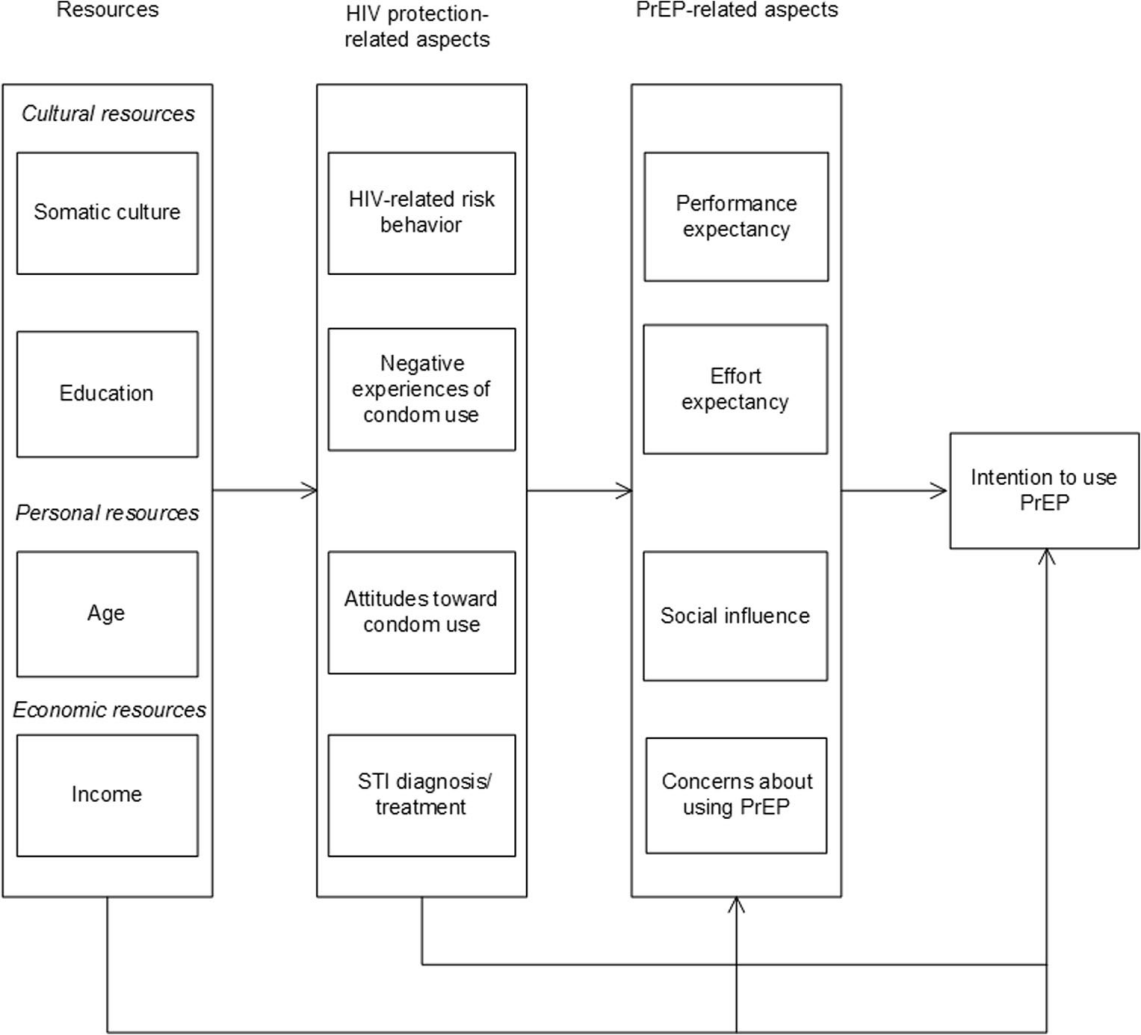
The hypothetical model explaining the intention to use PrEP in MSM

The variables were systematized and configured in three groups: PrEP-related aspects, HIV protection-related aspects and the resources of MSM. This model is shown in Fig. 1.

Drawing on this model, we hypothesized that (1) the intention to use PrEP would be predicted directly by PrEP-related aspects, including performance expectancy, effort expectancy, social influence and concerns about using PrEP. We further hypothesized that (2) these variables would, in turn, be predicted by HIV protection-related aspects, including HIV-related risk behavior, negative experiences of condom use, attitudes toward condom use and STI diagnoses, and treatment in the previous 6 months, which might also have direct effects on the intention to use PrEP. Finally, we surmised that (3) the resources of MSM, namely, somatic culture, education, age and income, would predict HIV and PrEP-related aspects and might also have a direct effect on the intention to use PrEP.

## Methods

### Design and procedure

The study design was cross-sectional. We collected the data by means of an anonymous, standardized self-administered questionnaire. The questionnaire was made available either online or in a paper-and-pencil format. The online version was constructed using ‘EFS Survey’ (Quest Back AG) and made accessible with an open access link that could be distributed and redistributed using any communication media. The paper-and-pencil version was made available through the study-specific website.

The questionnaire included a short description of PrEP drawing on the publications of the US Centers of Disease Control and Prevention (2014). It provided information on the application, effectiveness and potential side effects of a regime of daily oral PrEP and provided a picture of the tablet alongside a scale bar. Two experts reviewed the description: the manufacturer’s local Senior Medical Manager and an HIV specialist involved in the Swiss HIV Cohort Study.

We tested the questionnaire in a pre-test with 32 MSM to verify its comprehensibility and usability and gathered feedback from survey experts and medical professionals. As a result of the feedback, we slightly adjusted the wording of the questionnaire. The internal consistency of all scales proved to be acceptable.

Three identical versions of the questionnaire (German, French and Italian) were prepared. The first version was in German. To obtain equivalent French and Italian versions, the German questionnaire was translated into French and Italian, with the outcome being verified using a back-translation procedure. It took approximately 25 min to complete.

Informed consent was obtained from all individual participants included in the study. The questionnaire included information about the study and instructions, the affirmation that participation was voluntary, a confirmation of anonymity and the participant’s consent that they agreed to the inclusion of their data in the analysis. We also set up a website providing information about the study and offered the opportunity to contact the research team if there were any questions or a need for further information. All data were treated confidentially; only the research team had access to the data.

We recruited participants through different channels: We set up profiles on several online dating platforms designed for MSM, posted on social media, distributed flyers and posters in bars and saunas where MSM meet, displayed material at events and parties organized by and for gay and bisexual men and placed information in gay-specific sexual health clinics. In addition, we drew attention to our study by publishing interviews about the project in local gay magazines and newspapers. From May 2015 to December 2015, we gathered a convenience sample of 659 HIV-negative MSM living in Switzerland. Participants who reported being HIV-positive or of unknown HIV status, those who were not living in Switzerland, did not answer the question about the intention to use PrEP or did not give their consent were excluded from the sample. In total, 556 participants were included into the final analyses.

### Measures

The measures of the model variables are listed in Table 1. Drawing from previous tests of the UTAUT (Holden and Karsh 2010; Oye et al. 2014; Venkatesh et al. 2003), we used the intention to use PrEP to assess the acceptability of PrEP. To measure the PrEP-related aspects stemming from the UTAUT, validated instruments were used (Holden and Karsh 2010; Oye et al. 2014; Venkatesh et al. 2003). The concerns about PrEP were assessed using a formative measure (Ayala et al. 2013; Golub et al. 2013). HIV risk behavior was measured by the proportion of unprotected anal intercourse with casual partners during the previous 6 months ranging from 0 to 100% (Kalichman et al. 2005; Nideröst et al. 2011). To assess the negative experiences of condom use, six items were used in accordance with the results from a previous study (Gredig et al. 2016). Attitudes toward condoms were measured drawing on Reece et al. (2010), using ten items on a 7-point semantic differential scale (Porst 2011). Having experienced sexually transmitted infections (STI) was operationalized as having been diagnosed and treated for an STI during the previous 6 months (Nideröst et al. 2011). Resources included the respondents’ age, their formal level of education, their personal income per month and the type of somatic culture they had adopted. We used measures adapted to the Swiss context to capture formal levels of education and income (Federal Statistical Office 2008, 2015). To identify the type of somatic culture, we used a formative index including 22 items expressing orientations in dealing with the body that had been validated in prior research (Gredig et al. 2007). In men living in Switzerland, four types of somatic cultures have been identified: the visionary, ambivalent, functionalistic and ‘easy-going’ type of somatic culture (see Gredig et al. (2002) for a detailed description).

**Table 1:** Measures of the model variables

Variable	Number of items	Response scale	Range of calculated scores	Cronbach’s alpha	Source
Acceptability					
Intention to use PrEP ‘How likely are you to use PrEP when available in Switzerland?’	1	Seven-point Likert scale ranging from 1 = ‘very unlikely’ to 7 = ‘very likely’	1–7		
PrEP-related aspects					
Performance expectancy e.g., ‘I could live my sexuality in a less complicated way’	11	Seven-point Likert scale ranging from 1 = ‘completely disagree’ to 7 = ‘completely agree’	1–7	0.91	(Holden and Karsh 2010; Oye et al. 2014; Venkatesh et al. 2003)
Effort expectancy e.g., ‘Taking a pill every day would be easy for me’	8	Seven-point Likert scale ranging from 1 = ‘completely disagree’ to 7 = ‘completely agree’	1–7	0.75	(Holden and Karsh 2010; Oye et al. 2014; Venkatesh et al. 2003)
Social influence e.g., ‘People who are important to me would recommend that I use PrEP’	4	Seven-point Likert scale ranging from 1 = ‘completely disagree’ to 7 = ‘completely agree’	1–7	0.92	(Holden and Karsh 2010; Oye et al. 2014; Venkatesh et al. 2003)
Concerns about using PrEP e.g., ‘I’m worried about negative long-term effects’ or ‘People in my environment could think I was HIV positive’	17	Seven-point Likert scale ranging from 1 = ‘completely disagree’ to 7 = ‘completely agree’	1–7	0.86	(Ayala et al. 2013; Golub et al. 2013)
HIV protection-related aspects					
Proportion of unprotected anal intercourse with casual partners 6 months prior to survey	1		0–100		(Kalichman et al. 2005; Nideröst et al. 2011)
Negative experiences of condom use e.g., ‘The condom slipped off’	6	Seven-point Likert scale ranging from 1 = ‘never’ to 7 = ‘always’	1–7	0.64	(Gredig et al. 2016)
Attitudes toward condoms e.g., ‘It is a relief to use a condom’ and ‘Using a condom is boring’	10	Seven-point semantic differential scale ranging from −3 to +3	1–7	0.89	(Reece et al. 2010)
Being diagnosed and treated for an STI 6 months prior to survey	1	Yes/no	0–1		(Nideröst et al. 2011)
Resources					
Age in years	1		15–78		
University or University of Applied Sciences Degree	1	Yes/no	0–1		(Federal Statistical Office 2008)
Personal income in CHF	1	1 = Less than 13,000 2 = 13,001–26,000 3 = 26,001–39,000 4 = 39,001–52,000 5 = 52,001–65,000 7 = 78,001–91,000 8 = 91,001–104,000 9 = 104,001–127,000 10 = 127,001–150,000 11 = 150,001–175,000 12 = more than 175,001	1–12		(Federal Statistical Office 2015)
Somatic culture	1	1 = visionary type 2 = ambivalent type 3 = functionalistic type 4 = easy-going type	–		(Gredig et al. 2007; Gredig et al. 2002)

### Data analysis

Data were imported from the EFS-Survey into IBM SPSS 22. First, we performed descriptive statistics by conducting frequency analysis and described the results using central tendency, dispersion (M, SD) and distribution where appropriate. Second, we determined the correlations of model variables using bivariate and multivariate analyses. Third, the hypothesized causal paths were analyzed using structural equation modeling. Variables that were measured on a nominal level, such as the type of somatic culture adopted and the level of formal education, were transformed into dummy variables. As structural equation modeling requires complete data sets and given that 14 variables were implied, missing data could have entailed the exclusion of a number of participants and potential bias. To avoid exclusions, we performed a multiple imputation as suggested by Lüdtke et al. (2007). Structural equation modeling was performed using the Generalized Least Square Estimates method in AMOS 22. All variables were entered into the equation at once and paths were considered according to the results of the previous multivariate analyses.

## Results

### Sample description

The mean age of the participants was 40.5 years (*SD* = 11.9) ranging from 15 to 81 years. About 43.7% (*n* = 243) of the respondents reported being single, while 50.9% (*n* = 283) were in a steady relationship with a man and 5.7% (*n* = 32) were in a steady relationship with a woman. The mean duration of these relationships was 8.6 years (*SD* = 7.6), ranging from 1 month to 35 years. A proportion of 13.5% (*n* = 38) of the participants reported having a seropositive steady partner; 82.6% (*n* = 232) reported having a seronegative partner, and 3.9% (*n* = 11) reported being unaware of their partner’s serostatus. Regarding the type of somatic culture adopted, 30.1% (*n* = 167) of the participants identified with a visionary, 22.7% (*n* = 126) with an ambivalent, 24.3% (*n* = 135) with a functionalistic and 22.9% (*n* = 127) with an ‘easy-going’ type of somatic culture. Table 2 displays further sociodemographic characteristics and resources of the sample.

**Table 2:** Socio-demographic characteristics and resources of participants (N = 556)

Variable	N	%
Sexual orientation (*N* = 555)		
Homosexual, gay	489	88.1
Bisexual	62	11.2
Pansexual	2	0.4
Other	2	0.4
Formal education (N = 555)		
Primary school	1	0.2
Compulsory education	14	2.5
Grammar school, high school, vocational baccalaureate college	43	7.7
Teacher training college	5	0.9
Apprenticeship, college of trade and industry (full-time)	110	19.8
Advanced professional training	78	14.0
Higher professional college	58	10.4
University/university of applied sciences	246	44.3
Employment situation ^a^ (N = 556)		
Full-time employment	382	68.7
Part-time employment	93	16.7
University studies	48	8.6
Unemployed	14	2.5
Retired	20	3.6
Unable to work	10	1.8
Performing housework	3	0.5
Self-employed	7	1.3
Other	5	0.9
Income in CHF (*N* = 546)		
Less than 13,000	23	4.2
13,000–26,000	20	3.7
26,001–39,000	33	6.0
39,001–52,000	37	6.8
52,001–65,000	36	6.6
65,001–78,000	64	11.7
78,001–91,000	66	12.1
91,001–104,000	78	14.3
104,001–127,000	70	12.8
127,001–150,000	53	9.7
150,001–175,000	25	4.6
More than 175,000	41	7.5
Area of residence/population size (N = 555)		
Rural area	123	22.2
Small town (less than 20,000 inhabitants)	91	16.4
Town (21,000–100,000 inhabitants)	73	13.2
City (more than 100,000 inhabitants)	268	48.3
Language (N = 556)		
German speaking	441	79.3
French speaking	109	19.6
Italian speaking	6	1.1
Country of birth (N = 555)		
Switzerland	428	77.1
Other	127	22.9

Note: ^a^ multiple answers possible

### Intention to use PrEP

In our sample, the intention to use PrEP was moderate. About 12.9% (*n* = 72) of the participants reported that they were very likely to use PrEP when available, and 26.4% (*n* = 147) reported they were likely to somewhat likely to do so. Almost 20.5% (*n* = 114) were very unlikely to use it, and 30.4% (*n* = 169) were unlikely to somewhat unlikely to use it. Only 9.7% (*n* = 54) were undecided. The mean score of intention to use PrEP was 3.7 (SD = 2.1) on a scale ranging from 1 to 7 (see also Table 3).

**Table 3:** Means and standard deviations of intention to use PrEP, PrEP-related and HIV protection-related aspects (N = 556)

	Mean	Standard deviation
Intention to use PrEP	3.7	2.11
PrEP-related aspects		
Performance expectancy (1–7)	4.4	1.41
Effort expectancy (1–7)	3.2	1.12
Social influence (1–7)	3.3	1.67
Concerns (1–7)	3.8	1.12
HIV protection-related aspects		
Negative experiences of condom use (1–7)	2.0	0.80
Attitudes toward condom use (1–7)	4.5	1.13
HIV risk behavior (0–100)	19.1	31.85
Treated for an STI (0/1)	0.2	0.37

### PrEP-related and HIV protection-related aspects

The means and standard deviations of PrEP-related and HIV protection-related aspects are shown in Table 3. A proportion of 83.5% of the participants (*n* = 464) reported a sexual encounter with at least one casual partner during the previous 6 months. The median number of casual partner in the previous 6 months reported was 7 (*IQR* = 3–14), the median number of occasions on which they had sexual intercourse with casual partners was 12 (*IQR* = 5–24). The HIV risk behavior (i.e., condomless sex) was moderate. Among those having had sex with a casual partner, 53% (*n* = 239) reported consistent condom use in all these encounters, while 9.5% (*n* = 43) reported no condom use at all. About 16.4% (*n* = 91) reported having been treated for an STI in the previous 6 months. The most frequent diagnoses were gonorrhea (31.9%; *n* = 29), chlamydia (28.6%; *n* = 26) and syphilis (20.9%; *n* = 19).

Regarding the negative experiences with condoms, only 16.8% (*n* = 93) reported having difficulties in the use of condoms (scores between 4 and 7). About 51% (*n* = 282) had a positive attitude toward condoms (scores between 5 and 7).

### Test of the model

The analysis revealed that participants’ intention to use PrEP was predicted by the four PrEP-related aspects: performance expectancy (β = 0.25), effort expectancy (β = −0.19), social influence (β = 0.31) and concerns (β = −0.15). Furthermore, it is, although less strongly, predicted by HIV protection-related aspects: attitudes toward condom use (β = −0.07) and negative experiences of condom use (β = 0.09). Additionally, one of the resources, respondents’ age (β = −0.09) was a predictor of the intention to use PrEP. The strongest predictor, however, was social influence.

These variables, in turn, were predicted by variables conveying HIV protection-related aspects and resources. Thus, performance expectancy was predicted by negative experiences of condom use (β = 0.18), attitudes toward condom use (β = −0.21), age (β = −0.08) and income (β = 0.14). Having been treated for an STI in the previous 6 months (β = 0.08) was not a significant predictor. The strongest predictor was attitudes toward condom use. Effort expectancy was predicted by the income (β = −0.15), age (β = −0.10) and having adopted an ambivalent type of somatic culture (β = −0.08). The strongest predictor was income. Social influences were predicted by risk behavior in the 6 months prior to the survey (β = 0.08), negative experiences of condom use (β = 0.23) and income (β = 0.10). Attitudes toward condom use (β = −0.07) were not significant. The strongest predictor was negative experiences of condom use. Lastly, concerns were predicted by attitudes toward condom use (β = −0.09), having been treated for an STI in the previous 6 months (β = −0.08), having adopted a visionary type of somatic culture (β = −0.08) and the level of formal education (β = −0.10). All of these predictors were rather weak. The strongest was the level of formal education.

Regarding these HIV protection-related aspects, analysis shows that the level of HIV risk behavior in the 6 months prior to the survey was predicted by formal education (β = −0.10) while negative experiences of condom use were predicted by having adopted a visionary type of somatic culture (β = −0.11) and by the level of formal education (β = −0.10). Having been treated for an STI in the previous 6 months was predicted by age (β = −0.12).

**Fig. 2:**
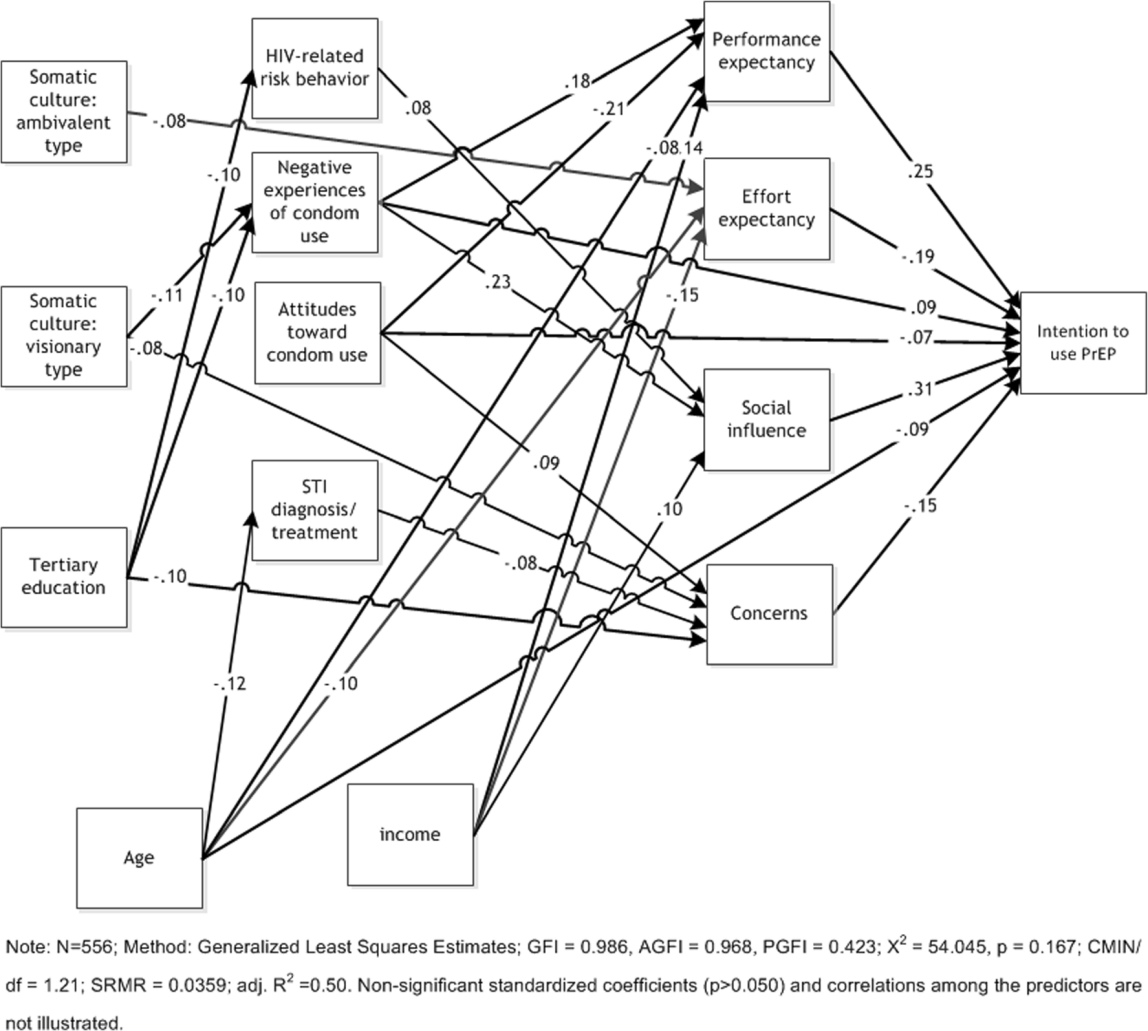
Visualization of the results of structural equation modeling. Note: *N* = 556; method: generalized least squares estimates; GFI = 0.986, AGFI = 0.968, PGFI = 0.423; X^2^ = 54.045, *p* = 0.167; CMIN/df = 1.21; SRMR = 0.0359; adj. R^2^ = 0.50. Non-significant standardized coefficients (*p* > 0.050) and correlations among the predictors are not illustrated

The model showed a good fit (GFI = 0.986, AGFI = 0.968) and proved to be satisfactorily parsimonious (PGFI = 0.423). The tested model explained 50.3% of the variance of the intention of MSM to use PrEP when available. The standardized regression weights implied are available in Table 4. Figure 2 shows the path diagram of the final model explaining the intention to use PrEP.

**Table 4:** Unstandardized, standardized and significance levels for model in Fig. 2 (standard errors in parentheses; N = 556)

Parameter estimate			Unstandardized	Standardized	*p*
Experiences of condom use	<−--	Somatic culture: visionary type	−0.19 (0.07)	−0.11	0.004
Experiences of condom use	<−--	Tertiary education	−0.15 (0.06)	−0.10	0.012
Risk behavior	<−--	Tertiary education	−6.41 (2.57)	−0.10	0.012
Treated for an STI	<−--	Age	−0.00 (0.00)	−0.12	0.004
Effort expectancy	<−--	Age	−0.01 (0.00)	−0.10	0.011
Concerns	<−--	Attitudes toward condom use	0.09 (0.04)	0.10	0.018
Social influence	<−--	Attitudes toward condom use	−0.11 (0.06)	−0.08	0.077
Performance expectancy	<−--	Attitudes toward condom use	−0.27 (0.05)	−0.21	≤0.001
Performance expectancy	<−--	Negative experiences of condom use	0.33 (0.07)	0.18	≤0.001
Social influence	<−--	Negative experiences of condom use	0.49 (0.09)	0.23	≤0.001
Performance expectancy	<−--	Age	−0.01 (0.00)	−0.08	0.029
Social influence	<−--	Risk behavior	0.00 (0.00)	0.08	0.036
Performance expectancy	<−--	Treated for an STI	0.25 (0.14)	0.07	0.063
Concerns	<−--	Treated for an STI	−0.25 (0.12)	−0.08	0.032
Performance expectancy	<−--	Income	0.33 (0.07)	0.14	≤ 0.001
Effort expectancy	<−--	Income	−0.06 (0.02)	−0.15	≤ 0.001
Social influence	<−--	Income	0.06 (0.02)	0.10	0.015
Concerns	<−--	Tertiary education	−0.23 (0.09)	−0.10	0.008
Effort expectancy	<−--	Somatic culture: ambivalent type	−0.22 (0.10)	−0.08	0.025
Concerns	<−--	Somatic culture: visionary type	−0.20 (0.10)	−0.08	0.043
Intention to use	<−--	Performance expectancy	0.36 (0.06)	0.25	≤ 0.001
Intention to use	<−--	Effort expectancy	−0.37 (0.07)	−0.19	≤ 0.001
Intention to use	<−--	Social influence	0.39 (0.05)	0.31	≤ 0.001
Intention to use	<−--	Concerns	−0.28 (0.06)	−0.15	≤ 0.001
Intention to use	<−--	Attitudes toward condom use	−0.13 (0.06)	−0.07	0.039
Intention to use	<−--	Age	−0.02 (0.01)	−0.10	0.003
Intention to use	<−--	Negative experiences of condom use	0.23 (0.09)	0.09	0.013

*Note:* N = 556; method: generalized least squares estimates; GFI = 0.99, AGFI = 0.97, PGFI = 0.42; χ^2^ = 54.05, *p* = 0.17; CMIN/df = 1.21; SRMR = 0.04; adj. R^2^ = 0.50

## Discussion

These results can be discussed from various perspectives. First, from an HIV-prevention perspective, we acknowledge that the respondents’ intention to use PrEP was on a moderate level. This was also the case for the HIV-negative MSM with casual partners in our sample (*M =* 3.81, *SD =* 2.12). Overall, 12.9% were very likely and 26.4% were likely or somewhat likely to use PrEP when available. Thus, together, 39.3% reported some intention to use PrEP in the future.

Thus, in our sample, the proportion of MSM intending to use PrEP was smaller than in the sample of the brief survey comprising MSM living in Switzerland with a profile on the online dating platform Grindr®. Among those MSM, 49.9% reported an intention to use PrEP (Hampel et al. 2017). Our findings approximate those on MSM living in Australia before PrEP became available. In 2012, 10.7% of the respondents of the Australian study were very likely and 26% likely to use PrEP when it became available (Prestage et al. 2014).

Second, we would like to draw attention to the model tested. Regarding the variables drawn from the Unified Theory of Acceptance and Use of Technology (UTAUT), the analysis confirmed the performance expectancy, effort expectancy and social influence as predictors of the intention to use the new prevention technology. Notably, social influence turned out to be the strongest predictor, clearly outperforming the others. The UTAUT posits that age moderates these variables. In our study, age predicted the intention to use PrEP directly.

The analysis also confirmed that concerns were a predictor of the intention to use PrEP. In this way, the data from this investigation found support for the inclusion of this variable drawn from research regarding the willingness to use PrEP. Finally, it is noteworthy that two HIV protection-related aspects—attitudes toward condom use and negative experiences of condom use—also directly predicted the intention to use PrEP. Although their effects were small, they draw attention to the fact that the intention to use the new option in HIV prevention is linked with attitudes toward and experience of the use of a preceding and concurrent method.

The subsequent analysis not only confirmed the inclusion of HIV protection-related aspects but also considered participants’ resources. Indeed, the PrEP-related aspects were largely determined by the HIV protection-related aspects and resources. The resources predicted variables on three tiers of the model: Age predicted not only an HIV protection-related aspect (having been treated for an STI in the previous 6 months) but also PrEP-related aspects (performance expectancy and effort expectancy) and, directly, the intention to use PrEP. Income predicted directly and exclusively PrEP-related aspects (performance expectancy, effort expectancy and social influence). The level of formal education predicted a PrEP-related aspect (concerns) and two HIV protection-related aspects (HIV risk behavior and negative experiences of condom use). Having adopted a visionary type of somatic culture predicted a PrEP-related aspect (concerns) and an HIV protection-related aspect (experiences of condom use) while having adopted an ambivalent type of somatic culture predicted a PrEP-related aspect (effort expectancy). This depicts findings of previous research evidencing that men having adopted a visionary or ambivalent type of somatic culture were likely to integrate condom use: In both types condom use proved compatible with the orientations guiding the specific way to deal with their body (Gredig et al. 2002; Nideröst 2007). In sum, the data from this investigation support the hypothesis that all variables added to the model contribute to significant causal paths leading to the intention to use PrEP.

Third, we reflect on the use of the UTAUT. As highlighted above, the variables from the UTAUT predicted the intention to use PrEP. This suggests that the basic assumption of this model designed to explain acceptance of technology in general is also applicable to HIV chemoprophylaxis. Concomitantly, the test of the expanded model showed that it was worth extending the model and including PrEP specific variables (specific concerns about PrEP) as well as variables that express issues specific to HIV protection. These variables directly contribute to the explained variance in the intention to use PrEP.

The advantage of extending and contextualizing the model was, however, that it permitted the identification of various causal paths leading (1) from personal and economic as well as socio-cultural resources formed in the socialization process, through (2) HIV protection-related attitudes, experiences, views and behaviors (implying risks and respondents’ ways of forming relationships) and (3) the participants’ rating of the new prevention methods, to (4) their intention to use PrEP.

The findings also demonstrated the importance of acknowledging and considering the complexities of decision making for MSM on PrEP use. Our tested model also took account of the fact that, in the present situation, PrEP would not only be made known and—in the future might be made available—to MSM who were at the onset of their active sexual life and would have to define a personal HIV protection strategy for the first time in their lives. Rather, PrEP would be introduced to MSM who largely began to define their ways of dealing with the challenge and risk of infection from HIV some time ago, often even decades ago. Against this background, the model implied—and was confirmed in this respect—that MSM, in particular those with a longer sexual biography, would gauge the new prevention option in the light of the personal HIV-protection strategies they adopted, consider their experiences and evaluate PrEP use against their sexual trajectories and perceived risks (regarding the latter see, for example, Imhof et al. (2014). From the perspective of those with positive attitudes to condom use, who had experienced few problems with it, it might make sense to maintain the condom use with which they were comfortable and abstain from PrEP use.

### Limitations

This study had several limitations. The data are self-reported, which may entail a bias regarding the reported levels of sexual risk behavior and condom use, although evidence has relativized this concern (Weinhardt et al. 1998). Regarding the scales used, the internal consistency of the measure of negative experiences of condom use was not fully satisfactory (Cronbach’s alpha = 0.64). Furthermore, the study was based on a convenience sample of MSM, and the findings can only be generalized with considerable caution. However, given that the size and the characteristics of the MSM population are not known (Marcus et al. 2009), more stringent sampling strategies are not applicable. Nevertheless, the high proportion of participants holding a higher educational degree could entail a bias although it parallels findings from other studies on MSM in Switzerland (Lociciro et al. 2012). The number of respondents from the Italian-speaking area is small and reflects difficulties in reaching these MSM that is also known from prior research and prevention practice. Still, the sample was diverse and included MSM from various recruiting sites. Lastly, as PrEP is still unavailable in Switzerland, this study does not inform about the intention to use it under real-life conditions. Moreover, given the gap between intention and use, conclusions about future use would have to be drawn with caution.

## Conclusion

As the first of its kind, the present study on the intention to use PrEP provides insight into the complex dynamic underlying the intention to use this new prevention option among MSM in Switzerland. It takes a significant step beyond describing those willing to use PrEP and contributes to the development of an explanatory model of PrEP uptake while connecting to the research on the acceptability of new technology in other fields.

The findings also provide a more tangible result as they provide clues to an advanced understanding of the decision making of MSM regarding PrEP. They provide health professionals, prevention providers, HIV/AIDS service organizations and activists with an idea of the backgrounds and dynamics within which the decision to use PrEP is embedded. When drawing on both the theoretical and practical conclusions from these findings, we have to be aware of the context the men under investigation are living in. In Switzerland, MSM have been addressed specifically by a prevention campaign with wide coverage that has continued to recommend condom use since its beginning 30 years ago (Kocher 1993). In a different context, the interplay of HIV protection-related and PrEP-related aspects could be different. Nevertheless, the present model may serve as a basis for further investigation into the explanation of the intention to use PrEP.

For health promotion and prevention, it would be important to make PrEP available for MSM at high risk for HIV infection. Considering that a supportive social influence, high performance expectancy as well as low effort expectancy and low concerns were the main predictors increasing the intention to use PrEP in the surveyed MSM, public health stakeholders should broader inform prospective users about this prevention option. For example, PrEP could be promoted as an additional prevention method within a combined HIV-prevention approach (condom use and treatment as prevention) in prevention campaigns and personal counseling addressing especially HIV-negative MSM with a negative attitude toward condoms or who have experienced problems with condom use in the past. For those MSM who have had little problem with condom use, there might be good reasons to maintain the condom use with which they were comfortable and to abstain from PrEP use. Thus, the promotion of PrEP should be carefully worded so as not to withdraw the legitimacy of condom use within the MSM community and should continue supporting condom use as an eligible prevention option.

## References

[CR1] Aghaizu A, Mercey D, Copas A, Johnson AM, Hart G, Nardone A (2013). Who would use PrEP? Factors associated with intention to use among MSM in London: a community survey. Sex Transm Infect.

[CR2] Ajzen I (1991). The theory of planned behavior: some unresolved issues. Organ Behav Hum Decis Process.

[CR3] Auerbach JD, Hoppe TA (2015). Beyond “getting drugs into bodies”: social science perspectives on pre-exposure prophylaxis for HIV. J Int AIDS Soc.

[CR4] Ayala G, Makofane K, Santos G-M, Beck J, Do TD, Hebert P, Wilson PA, Pyun T, Arreola S (2013). Access to basic HIV-related services and PrEP acceptability among men who have sex with men worldwide: barriers, facilitators, and implications for combination prevention. Sex Transm Dis.

[CR5] Baeten JM, Donnell D, Ndase P, Mugo NR, Campbell JD, Wangisi J, Tappero JW, Bukusi EA, Cohen CR, Katabira E, Ronald A, Tumwesigye E, Were E, Fife KH, Kiarie J, Farquhar C, John-Stewart G, Kakia A, Odoyo J, Mucunguzi A, Nakku-Joloba E, Twesigye R, Ngure K, Apaka C, Tamooh H, Gabona F, Mujugira A, Panteleeff D, Thomas KK, Kidoguchi L, Krows M, Revall J, Morrison S, Haugen H, Emmanuel-Ogier M, Ondrejcek L, Coombs RW, Frenkel L, Hendrix C, Bumpus NN, Bangsberg D, Haberer JE, Stevens WS, Lingappa JR, Celum C (2012). Antiretroviral prophylaxis for HIV prevention in heterosexual men and women. New Engl J Med.

[CR6] Barash EA, Golden M (2010). Awareness and use of HIV pre-exposure prophylaxis among attendees of a Seattle gay pride event and sexually transmitted disease clinic. AIDS Patient Care STDs.

[CR7] Beyrer C, Baral SD, van Griensven F, Goodreau SM, Chariyalertsak S, Wirtz AL, Brookmeyer R (2012). Global epidemiology of HIV infection in men who have sex with men. Lancet.

[CR8] Beyrer C, Bekker L-G, Pozniak A, Barré-Sinoussi F (2015). Pre-exposure prophylaxis works—it’s time to deliver. Lancet.

[CR9] Boltanski L, Kamper D, Rittner V (1976). Die soziale Verwendung des Körpers. Zur Geschichte des Körpers. Perspektiven der Anthropologie.

[CR10] Brooks RA, Kaplan RL, Lieber E, Landovitz RJ, Lee S-J, Leibowitz AA (2011). Motivators, concerns, and barriers to adoption of pre-exposure prophylaxis for HIV prevention among gay and bisexual men in HIV serodiscordant male relationships. AIDS Care.

[CR11] Brooks RA, Landovitz RJ, Kaplan RL, Lieber E, Lee S-J, Barkley TW (2012). Sexual risk behaviors and acceptability of HIV pre-exposure prophylaxis among HIV-negative gay and bisexual men in Serodiscordant relationships: a mixed methods study. AIDS Patient Care STDs.

[CR12] Bundesamt für Gesundheit (2008) HIV-Epidemie in der Schweiz 2007: Trends bestätigt. Bulletin (6):84–87

[CR13] Bundesamt für Gesundheit (2010) HIV/Aids in der Schweiz am 31. Dezember 2009. Bulletin (5):81–83

[CR14] Bundesamt für Gesundheit (2016) HIV und Aids in der Schweiz im Jahr 2015. BAG Bulletin (46):14–21

[CR15] Centers for Disease Control and Prevention (2014) Pre-exposure Prophylaxis (PrEP) for HIV Prevention. http://www.cdc.gov/hiv/pdf/PrEP_fact_sheet_final.pdf. Accessed 23 August 2017

[CR16] Davis FD (1989). Perceived usefulness, perceived ease of use, and user acceptance of information technology. MIS Q.

[CR17] Delaney KP, Sanchez T, Bowles K, Oraka E, DiNenno E, Sullivan P (2016) Awareness and Use of PrEP Appear to Be Increasing Among Internet Samples of US MSM. Paper presented at the Conference on Retroviruses and Opportunistic Infections (CROI), Boston, Massachusetts, February 22–25, 2016

[CR18] Ding Y, Yan H, Ning Z, Cai X, Yang Y, Pan R, Zhou Y, Zheng H, Gao M, Rou K, Wu Z, He N (2016). Low willingness and actual uptake of pre-exposure prophylaxis for HIV-1 prevention among men who have sex with men in shanghai, China. Biosci Trends.

[CR19] Eaton LA, Driffin DD, Smith H, Conway-Washington C, White D, Cherry C (2014). Psychosocial factors related to willingness to use pre-exposure prophylaxis for HIV prevention among black men who have sex with men attending a community event. Sex Health.

[CR20] Eaton LA, Driffin DD, Bauermeister J, Smith H, Conway-Washington C (2015). Minimal awareness and stalled uptake of pre-exposure prophylaxis (PrEP) among at risk, HIV-negative, black men who have sex with men. AIDS Patient Care STDs.

[CR21] Eisingerich AB, Wheelock A, Gomez GB, Garnett GP, Dybul MR, Piot PK (2012) Attitudes and acceptance of oral and parenteral HIV Preexposure prophylaxis among potential user groups: a multinational study. PLoS One 7(1):e28238. 10.1371/journal.pone.0028238 10.1371/journal.pone.0028238 PMC3256136 22247757

[CR22] Elst EM, Mbogua J, Operario D, Mutua G, Kuo C, Mugo P, Kanungi J, Singh S, Haberer J, Priddy F, Sanders EJ (2013). High acceptability of HIV pre-exposure prophylaxis but challenges in adherence and use: qualitative insights from a phase I trial of intermittent and daily PrEP in at-risk populations in Kenya. AIDS Behav.

[CR23] Federal Statistical Office (2008). International standard classification of education (ISCED 97).

[CR24] Federal Statistical Office (2015). SLFS 2015 in brief. The Swiss labour force survey.

[CR25] Frankis J, Young I, Flowers P, McDaid L (2016). Who will use pre-exposure prophylaxis (PrEP) and why?: understanding PrEP awareness and acceptability amongst men who have sex with men in the UK – a mixed methods study. PLoS One.

[CR26] Galea JT, Kinsler JJ, Salazar X, Lee S-J, Giron M, Sayles JN, Cáceres C, Cunningham WE (2011). Acceptability of pre-exposure prophylaxis as an HIV prevention strategy: barriers and facilitators to pre-exposure prophylaxis uptake among at-risk Peruvian populations. Int J STD AIDS.

[CR27] Golub SA, Gamarel KE, Rendina HJ, Surace A, Lelutiu-Weinberger CL (2013). From efficacy to effectiveness: facilitators and barriers to PrEP acceptability and motivations for adherence among MSM and transgender women in new York City. AIDS Patient Care STDs.

[CR28] Grant RM, Lama JR, Anderson PL, McMahan V, Liu AY, Vargas L, Goicochea P, Casapia M, Guanira-Carranza JV, Ramirez-Cardich ME, Montoya-Herrera O, Fernandez T, Veloso VG, Buchbinder SP, Chariyalertsak S, Schechter M, Bekker LG, Mayer KH, Kallas EG, Amico KR, Mulligan K, Bushman LR, Hance RJ, Ganoza C, Defechereux P, Postle B, Wang FR, McConnell JJ, Zheng JH, Lee J, Rooney JF, Jaffe HS, Martinez AI, Burns DN, Glidden DV, iPrEx Study Team (2010). Preexposure chemoprophylaxis for HIV prevention in men who have sex with men. New Engl J Med.

[CR29] Gredig D, Parpan A, Nideröst S (2002). Somatische Kultur und HIV-Schutzstrategien heterosexueller Männer. Soz Präventiv Med.

[CR30] Gredig D, Nideröst S, Parpan-Blaser A (2007). Explaining the condom use of heterosexual men in a high-income country: adding somatic culture to the theory of planned behaviour. J Public Health.

[CR31] Gredig D, Uggowitzer F, Hassler B, Weber P, Nideröst S (2016). Acceptability and willingness to use HIV pre-exposure prophylaxis among HIV-negative men who have sex with men in Switzerland. AIDS Care.

[CR32] Grov C, Whitfield THF, Rendina HJ, Ventuneac A, Parsons JT (2015). Willingness to take PrEP and potential for risk compensation among highly sexually active gay and bisexual men. AIDS Behav.

[CR33] Hampel B, Kusejko K, Braun DL, Harrison-Quintana J, Kouyos R, Fehr J (2017) Assessing the need for a pre-exposure prophylaxis programme using the social media app Grindr(R). HIV Med:1–5. 10.1111/hiv.12521 10.1111/hiv.12521 PMC5655778 28544123

[CR34] Holden RJ, Karsh B-T (2010). The technology acceptance model: its past and its future in health care. J Biomed Inform.

[CR35] Holt M, Murphy DA, Callander D, Ellard J, Rosengarten M, Kippax SC, de Wit JBF (2012). Willingness to use HIV pre-exposure prophylaxis and the likelihood of decreased condom use are both associated with unprotected anal intercourse and the perceived likelihood of becoming HIV positive among Australian gay and bisexual men. Sex Transm Infect.

[CR36] Hugo JM, Stall RD, Rebe K, Egan JE, De Swardt G, Struthers H, McIntyre JA (2016). Anti-retroviral therapy based HIV prevention among a sample of men who have sex with men in cape town, South Africa: use of post-exposure prophylaxis and knowledge on pre-exposure prophylaxis. AIDS Behav.

[CR37] Imhof C, Favre O, Gredig D (2014). Safer Sex und die “erste Generation HIV”. Schutzstrategien und Risikoverhalten von Männern, die Sex mit Männern haben.

[CR38] Kalichman S, Malow R, Dévieux J, Stein JA, Piedman F (2005). HIV risk reduction for substance using seriously mentally ill adults: test of the information-motivation-behavior skills (IMB) model. Community Ment Health J.

[CR39] Khawcharoenporn T, Kendrick S, Smith K (2012). HIV risk perception and Preexposure prophylaxis interest among a heterosexual population visiting a sexually transmitted infection clinic. AIDS Patient Care STDs.

[CR40] Kirby T, Thornber-Dunwell M (2014). Uptake of PrEP for HIV slow among MSM. Lancet.

[CR41] Kocher KW (1993). Die stop Aids-story 1987–1992.

[CR42] Krakower DS, Mimiaga MJ, Rosenberger JG, Novak DS, Mitty JA, White JM, Mayer KH (2012). Limited awareness and low immediate uptake of pre-exposure prophylaxis among men who have sex with men using an internet social networking site. PLoS One.

[CR43] Leonardi M, Lee E, Tan DHS (2011). Awareness of, usage of and willingness to use HIV pre-exposure prophylaxis among men in downtown Toronto, Canada. Int J STD AIDS.

[CR44] Liu AY, Cohen SE, Vittinghoff E, Anderson PL, Doblecki-Lewis S, Bacon O, Chege W, Postle BS, Matheson T, Amico KR, Liegler T, Rawlings MK, Trainor N, Blue RW, Estrada Y, Coleman ME, Cardenas G, Feaster DJ, Grant R, Philip SS, Elion R, Buchbinder S, Kolber MA (2016). HIV pre-exposure prophylaxis integrated with municipal and community based sexual health services. JAMA Intern Med.

[CR45] Lociciro S, Jeannin A, Dubois-Arber F (2012). Evaluation de la compagne Break The Chain 2012. Raport intermédiaire au 31 Juillet 2012.

[CR46] Lüdtke O, Robitzsch A, Trautwein U, Köller O (2007). Umgang mit fehlenden Werten in der psychologischen Forschung. Psychol Rundsch.

[CR47] Marcus U, Schmidt AJ, Kollan C, Hamouda O (2009). The denominator problem: estimating MSM-specific incidence of sexually transmitted infections and prevalence of HIV using population sizes of MSM derived from internet surveys. BMC Public Health.

[CR48] McCormack S, Dunn DT, Desai M, Dolling DI, Gafos M, Gilson R, Sullivan AK, Clarke A, Reeves I, Schembri G, Mackie N, Bowman C, Lacey CJ, Apea V, Brady M, Fox J, Taylor S, Antonucci S, Khoo SH, Rooney J, Nardone A, Fisher M, McOwan A, Phillips AN, Johnson AM, Gazzard B, Gill ON (2016). Pre-exposure prophylaxis to prevent the acquisition of HIV-1 infection (PROUD): effectiveness results from the pilot phase of a pragmatic open-label randomised trial. Lancet.

[CR49] Mimiaga MJ, Case P, Johnson CV, Safren SA, Mayer KH (2009). Pre-exposure antiretroviral prophylaxis (PrEP) attitudes in high risk Boston area MSM: limited knowledge and experience, but potential for increased utilization after education. J Acquir Immune Defic Syndr.

[CR50] Molina J-M, Capitant C, Spire B, Pialoux G, Cotte L, Charreau I, Tremblay C, Le Gall J-M, Cua E, Pasquet A, Raffi F, Pintado C, Chidiac C, Chas J, Charbonneau P, Delaugerre C, Suzan-Monti M, Loze B, Fonsart J, Peytavin G, Cheret A, Timsit J, Girard G, Lorente N, Préau M, Rooney JF, Wainberg MA, Thompson D, Rozenbaum W, Doré V, Marchand L, Simon M-C, Etien N, Aboulker J-P, Meyer L, Delfraissy J-F (2015). On-demand Preexposure prophylaxis in men at high risk for HIV-1 infection. New Engl J Med.

[CR51] Nideröst S (2007). Männer, Körper und Gesundheit. Somatische Kultur und soziale Milieus bei Männern.

[CR52] Nideröst S, Gredig D, Roulin C, Rickenbach M, the Swiss HIV Cohort Study, the Eurosupport 5 Study Group (2011). Predictors of HIV-protection behaviour in HIV-positive men who have sex with casual male partners: a test of the explanatory power of an extended information-motivation-Behavioural skills model. AIDS Care.

[CR53] Nodin N, Carballo-Diéguez A, Ventuneac AM, Balan IC, Remien R (2008). Knowledge and acceptability of alternative HIV prevention bio-medical products among MSM who bareback. AIDS Care.

[CR54] Oye ND, Iahad NA, Rahim NA (2014). The history of UTAUT model and its impact in ICT acceptance and usage by academicians. Educ Inform Tech.

[CR55] Peinado J, Lama JR, Galea JT, Segura P, Casapia M, Ortiz A, Montano SM, Kochel T, Sánchez J (2013). Acceptability of oral versus rectal HIV Preexposure prophylaxis among men who have sex with men and transgender women in Peru. J Int Assoc Provid AIDS Care.

[CR56] Peng P, Su S, Fairley CK, Chu M, Jiang S, Zhuang X, Zhang L (2017) A global estimate of the acceptability of pre-exposure prophylaxis for HIV among men who have sex with men: a systematic review and meta-analysis. AIDS Behav. 10.1007/s10461-017-1675-z 10.1007/s10461-017-1675-z 28176168

[CR57] Pérez-Figueroa RE, Kapadia F, Barton SC, Eddy JA, Halkitis PN (2015). Acceptability of PrEP uptake among racially/ethnically diverse young men who have sex with men: the P18 study. AIDS Educ Prev.

[CR58] Porst R (2011). Fragebogen. Ein Arbeitsbuch.

[CR59] Prestage GP, Holt M, Bavinton BR, Murphy D, Guy R, Bradley J, Zablotska I (2014) Motivations for PrEP use among non-HIV-positive Australian gay men. Poster THPE156. Paper presented at the 20th International AIDS Conference, Melbourne, 24.7.2014

[CR60] Reece M, Herbenick D, Hollub AV, Hensel DJ, Middlestadt SE (2010). A psychometric assessment of the multi-factor attitude toward condoms scale (MFACS). Int J Sex Health.

[CR61] Riddell J, Cohn JA (2016). Reaching high-risk patients for hiv preexposure prophylaxis. JAMA.

[CR62] Saberi P, Gamarel KE, Neilands TB, Comfort M, Sheon N, Darbes LA, Johnson MO (2012) Ambiguity, ambivalence, and apprehensions of taking HIV-1 pre-exposure prophylaxis among male couples in San Francisco: a mixed methods study. PLoS One 7(11):e50061. 10.1371/journal.pone.0050061 10.1371/journal.pone.0050061 PMC3498189 23166819

[CR63] Simpson L, Gumel AB (2017). Mathematical assessment of the role of pre-exposure prophylaxis on HIV transmission dynamics. Appl Math Comput.

[CR64] Smith DK, Toledo L, Smith DJ, Adams MA, Rothenberg R (2012). Attitudes and program preferences of African-American urban young adults about pre-exposure prophylaxis (PrEP). AIDS Educ Prev.

[CR65] Thigpen MC, Kebaabetswe PM, Paxton LA, Smith DK, Rose CE, Segolodi TM, Henderson FL, Pathak SR, Soud FA, Chillag KL, Mutanhaurwa R, Chirwa LI, Kasonde M, Abebe D, Buliva E, Gvetadze RJ, Johnson S, Sukalac T, Thomas VT, Hart C, Johnson JA, Malotte CK, Hendrix CW, Brooks JT (2012). Antiretroviral Preexposure prophylaxis for heterosexual HIV transmission in Botswana. New Engl J Med.

[CR66] UNAIDS (2014). The gap report.

[CR67] Venkatesh V, Morris MG, Gordon BD, Davis FD (2003). User acceptance of information technology: toward a unified view. MIS Quart.

[CR68] Vernazza PL, Graf I, Sonnenberg-Schwan U, Geit M, Meurer A (2011). Preexposure prophylaxis and timed intercourse for HIV-discordant couples willing to conceive a child. AIDS.

[CR69] Weinhardt LS, Forsyth AD, Carey MP, Jaworski BC, Durant LE (1998). Reliability and validity of self-report measures of HIV-related sexual behavior: progress since 1990 and recommendations for research and practice. Arch Sex Behav.

[CR70] World Health Organization (2014). Consolidated guidelines on HIV prevention, diagnosis, treatment and care for key populations.

[CR71] Yang D, Chariyalertsak C, Wongthanee A, Kawichai S, Yotruean K, Saokhieo P, Guadamuz T, Suwanvanichkij V, Beyrer C, Chariyalertsak S (2013). Acceptability of pre-exposure prophylaxis among men who have sex with men and transgender women in northern Thailand. PLoS One.

[CR72] Young I, McDaid L (2014). How acceptable are Antiretrovirals for the prevention of sexually transmitted HIV?: a review of research on the acceptability of oral pre-exposure prophylaxis and treatment as prevention. AIDS Behav.

[CR73] Zhang Y, Peng B, She Y, Liang H, Peng H, Qian H, Vermund S, Zhong X, Huang A (2013). Attitudes toward HIV pre-exposure prophylaxis among men who have sex with men in western China. AIDS Patient Care STDs.

